# A Rare Case of Sporadic Hemiplegic Migraine Mimicking Stroke: A Diagnostic Challenge Solved by Comprehensive History Taking

**DOI:** 10.7759/cureus.57790

**Published:** 2024-04-07

**Authors:** Ridhima Kaushal, Abhishek Kashyap, Sai Yogesh, Mokshita Agarwal, Indrajit Banerjee

**Affiliations:** 1 Internal Medicine, Government Multi Specialty Hospital, Chandigarh, IND; 2 Internal Medicine, Sir Seewosagur Ramgoolam Medical College, Belle Rive, MUS; 3 Emergency Department, Avadh Hospital & Heart Centre, Lucknow, IND; 4 Pharmacology, Sir Seewosagur Ramgoolam Medical College, Belle Rive, MUS; 5 Medicine, Sir Seewosagur Ramgoolam Medical College, Belle Rive, MUS

**Keywords:** sporadic hemiplegic migraine, hemiplegic migraine, migraine without aura, neurologic manifestations, migraine with aura, migraine disorders

## Abstract

Migraine, a common affliction, manifests as debilitating headaches often accompanied by auras. However, hemiplegic migraine presents an unusual symptomatology, inducing unilateral paralysis during attacks. This condition, occurring in two forms, familial and sporadic, merits attention due to its rarity. To raise awareness of this ailment, we recount the case of a 33-year-old woman. This instance serves as a poignant reminder of the potential severity and complexity of hemiplegic migraines. By shedding light on this less-understood variant, we aim to enhance recognition and understanding within medical communities and among the general public. Additionally, emphasizing the importance of thorough history taking in identifying characteristic features, such as the presence of auras or unilateral paralysis preceding headaches, is paramount. Understanding these nuances aids in accurate diagnosis and formulation of tailored management strategies. It's imperative to recognize the distinct characteristics of hemiplegic migraines to ensure timely and appropriate management for affected individuals, offering them relief and improving their quality of life.

## Introduction

Migraine is a common disorder that causes attacks of disabling headaches that can be accompanied by an aura in a third of patients [1]. Typically, a migraine aura has visual symptoms, but a hemiplegic migraine (HM) is a distinct condition in which there is motor and sensory weakness [2]. HM can be of two types: familial (FHM) and sporadic (SHM). FHM is an inherited autosomal dominant form of migraine, and genetic studies have shown the involvement of three genes, namely the *CACNA1A*, *ATP1A2*, and *SCN1A* genes. Mutations from these genes account for about 7-14% of FHM cases in some populations [3,4]. These individuals need to have a family history of migraine with aura [2]. However, if the patient presents with HM without any family history, it is termed SHM. Since there are no pathognomic laboratory or radiological findings, diagnosing HM is mainly based on clinical history and is challenging. Therefore, a good history of symptoms, identifying triggers, and family history can lead to an accurate diagnosis [2]. Mostly, neuroimaging is normal, and only in some cases, a few changes like cortical swelling, venous dilation, and cortical and meningeal enhancements are seen in the contralateral hemisphere to hemiparesis, which tends to disappear after the resolution of neurological deficits. Moreover, these outcomes are not exclusive to HM, making it challenging and difficult to only rely solely on imaging [3,5].

## Case presentation

A 33-year-old female patient presented to the emergency department with complaints of headache, right-sided weakness in both limbs, and vomiting. Three days before seeking care in the emergency department, she had experienced a severe throbbing pain on the right side of her head, which was acute in nature. The headache was accompanied by nausea. The headache was continuous, so she took paracetamol 500 mg, but the pain did not subside. In the past, she had similar episodes of headaches, which were accompanied by nausea. The next day, while having breakfast, she felt an “electric current” passing from the right side of her head to her right upper extremities. She said she experienced heaviness and numbness on the right side of her body, which was accompanied by a headache. There was no history of loss of consciousness, loss of speech, loss of vision, fever, trauma, or seizures preceding the episode. However, she also complained of a sensation of objects crawling over her right lower limbs. Two days after the onset of the patient's symptoms, the headache was continuous and was accompanied by nausea and vomiting. She had one episode of vomiting, which was enough to fill a bucket of 3L. Vomitus contained food contents. After that, she fell unconscious and was immediately rushed to the hospital.

Her past medical history included hypertension, which was diagnosed six months ago, for which she was taking amlodipine 5 mg/day, which she stopped two months prior to the current presentation. There was no history of cardiac disease, coagulation disorder, connective tissue disease, diabetes mellitus, smoking, alcohol, or any recreational drug abuse. As ascertained by personal interview, there was no history of similar complaints in her family members. On admission, vitals were in the normal ranges with 126/92 mmHg arterial blood pressure, 98.4°F temperature, saturation of peripheral oxygen (SpO2) 98%, 78/minute heart rate, and 20 breaths/minute respiratory rate. Intravenous cannula and Foley catheter were introduced, and the patient was monitored. A non-contrast computed tomography (CT) head and magnetic resonance imaging (MRI) were requested. General examination and examination of the chest, cardiovascular, and abdomen were unremarkable. On the central nervous system examination, she was drowsy and disoriented to time, place, and person. There was right hemiparesis with a power of muscle power scale ⅖ in her right upper and lower extremities with hemiplegic gait. The patient was given diclofenac, pantoprazole, ondansetron, enoxaparin, aspirin, atorvastatin, and alprazolam intravenously. 

**Figure 1:**
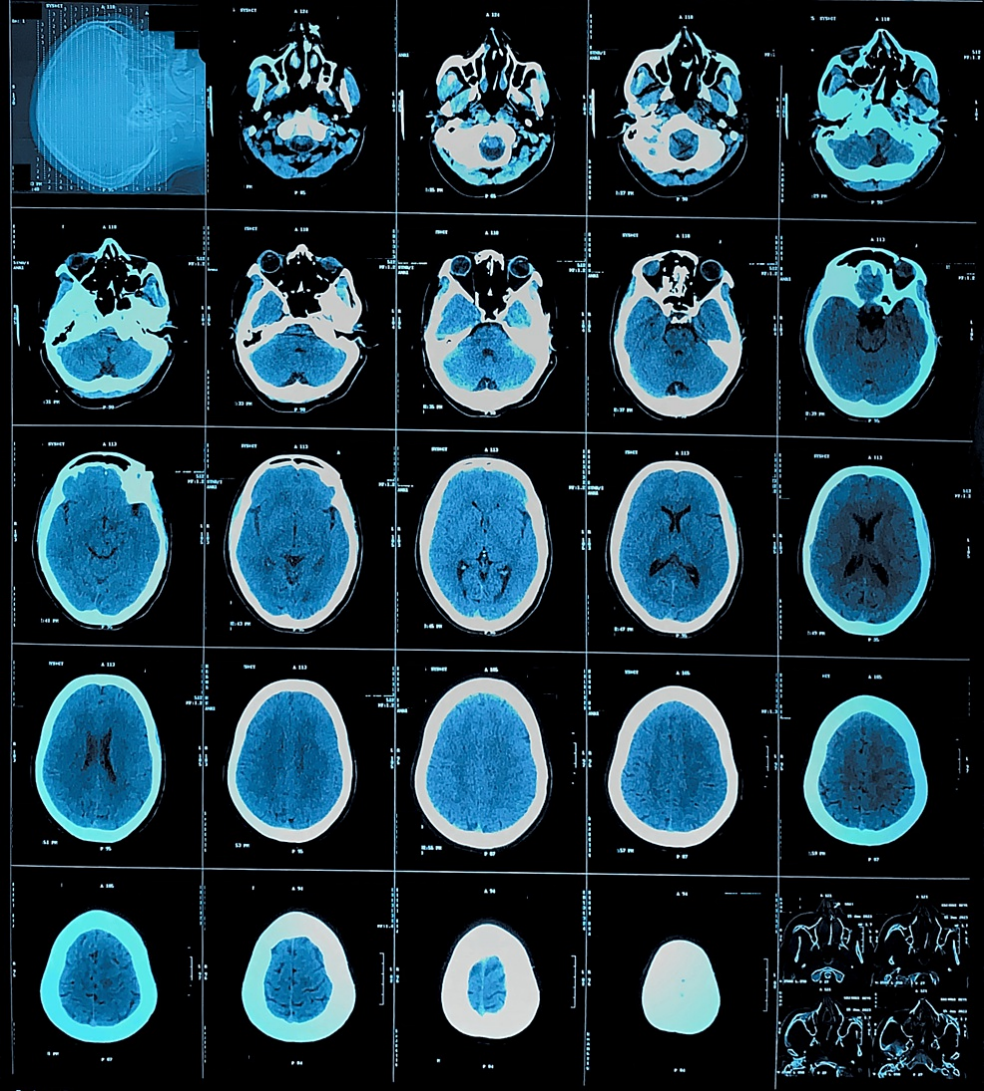
Plain Axial View CT Scan of Head

**Figure 2:**
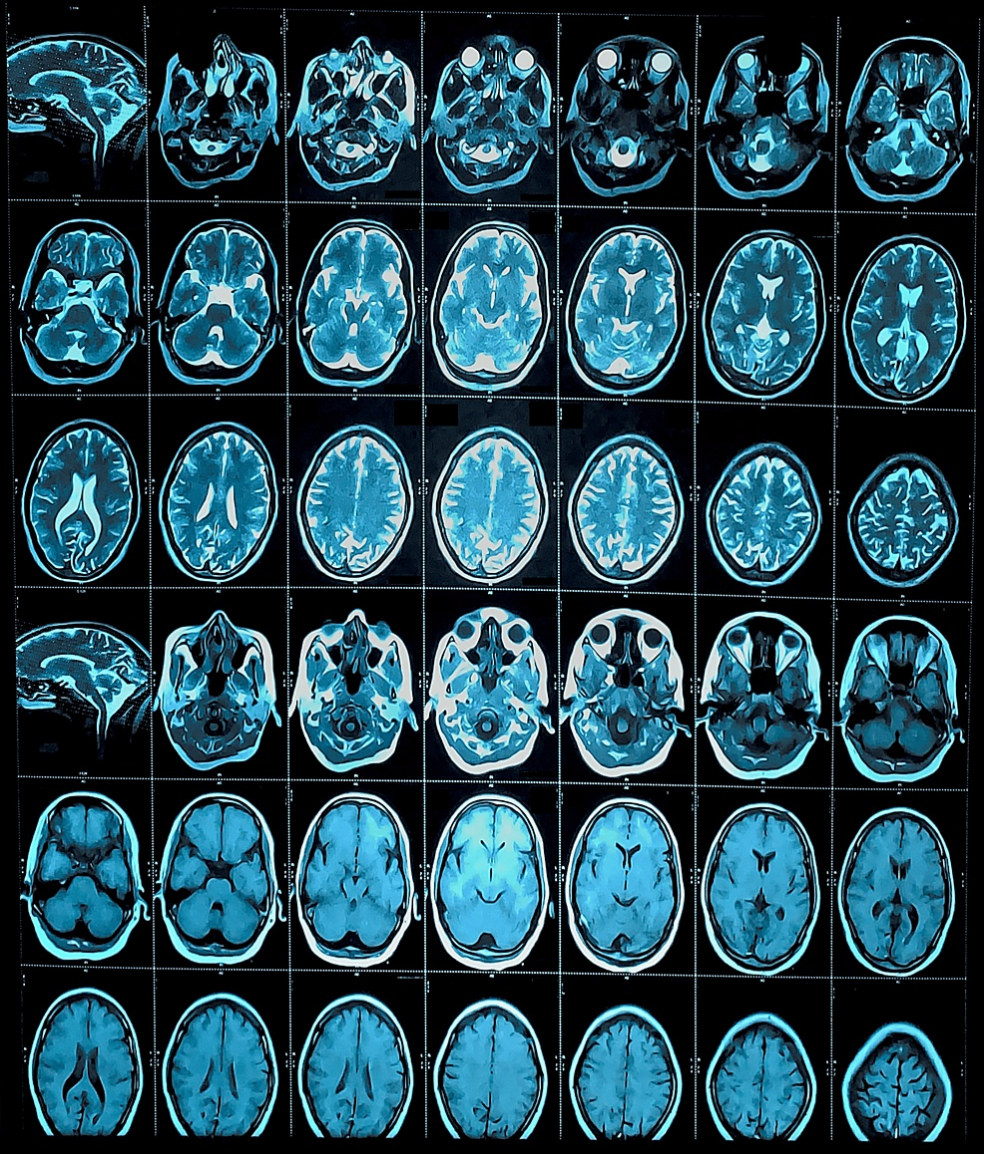
T2 Weighted Axial View MRI of the Brain

The following day, the muscle power scale was 3/5 in the right upper and lower limbs. Reflexes were brisk. Babinski's on the right side was mute, and the left side was plantar flexion. No bleeding was detected on the non-contrast CT head (Figure 1). No thrombus was detected in the MRI (Figure 2). Electrocardiography showed sinus bradycardia. Her hemogram, liver function tests, renal function tests, serum lipids, and thyroid function tests were within normal limits. Her international normalized ratio (INR) and prothrombin time (PT) were 4.50 and 59 seconds, respectively (Table 1). Genetic analysis for mitochondrial mutations could not be done. Two days later, the motor power scale of the right side was 4/5, and Babinski was mute. The patient’s motor and sensory complaints completely resolved after the third day of symptom onset. She was managed conservatively and made a gradual and complete recovery. Regrettably, the patient's inability to conduct a follow-up assessment was attributed to non-compliance.

**Table 1:** Laboratory Investigations

Test	Values	Normal Range
Fasting Blood Sugar	107 mg/dL	70-110 mg/dL
Haemoglobin	12.9 gm%	12.5-15gm%
Total Leukocyte Count	8200 cells/μL	4000-11000 cells/μL
Platelets	228000 platelets/μL	150,000-450,000 platelets/μL
Packed Cell Volume	36%	35-47%
Red Blood Cell Count	4.0 million cells/μL	4.6-6.0 million cells/μL
Total Bilirubin	1.7 mg/dL	0.1-1.2 mg/dL
Serum Glutamic Oxaloacetic Transaminase	61 unit/L	0-40 units/L
Serum Glutamic Pyruvic Transaminase	85 unit/L	0-40 unit/L
Alkaline Phosphatase	92 IU/L	20-110 IU/L
Serum Creatinine	0.8mg/dL	06-1.3mg/dL
Blood Urea	32mg/dL	14-45mg/dL
International Normalized Ratio (INR)	4.5 mg/dL	<=1.1 mg/dL
Prothrombin Time (PT)	59 seconds	10-13 seconds

## Discussion

According to the International Headache Society criteria for migraine with aura [6], the patient meets the criteria for SHM diagnosis (Table 2). Due to similar clinical features and complete reversibility, HM is often misdiagnosed as a transient ischemic attack (TIA). However, a detailed history can help differentiate between these conditions; TIA and stroke have a sudden onset, whereas HM has a gradual onset with aura. The timing of the headache is also essential since motor weakness precedes headache in HM, whereas headache follows weakness in hemorrhagic stroke. If the attack's duration is prolonged, it is often misdiagnosed as TIA [3].

**Table 2:** Diagnostic criteria by International Classification of Headache Disorders, 3rd edition (ICHD-3), which can help diagnose migraine with aura Reference: Headache Classification Committee of the International Headache Society (IHS) The International Classification of Headache Disorders, 3rd edition, 2018 [6]

	Criteria
A	At least 2 attacks fulfilling criteria B and C
B	One or more of the following fully reversible aura symptoms
	1. Visual
	2. Sensory
	3. Speech and/or language
	4. Motor
	5. Brainstem
	6. Retinal
C	At least three of the following six characteristics:
	1. at least one aura symptom spreads gradually over 5 minutes
	2. two or more aura symptoms occur in succession
	3. each individual aura symptom lasts 5–60 minutes
	4. at least one aura symptom is unilateral
	5. at least one aura symptom is positive
	6. the aura is accompanied or followed within 60 minutes, by a headache
D	Not better accounted for by another ICHD-3 diagnosis
Note:	
1.	When, for example, three symptoms occur during an aura, the acceptable maximal duration is 3x60 minutes. Motor symptoms may last up to 72 hours.
2.	Aphasia is always regarded as a unilateral symptom; dysarthria may or may not be.
3.	Scintillations and pins and needles are positive symptoms of aura

Research findings indicate that HM is associated with mutations in the alpha-1A subunit of the P/Q type neuronal calcium channel gene (*CACN1A1*) [7], resulting in higher cellular calcium influx; therefore, non-selective calcium channel blockers (CCB) such as verapamil inhibit this influx and modulate membrane excitability [8]. Since no particular evidence suggests that SHM should be treated differently than FHM, around 120mg of Oral Verapamil BD is helpful for treatment and prevention [9].

In individuals with progressive cerebellar ataxia, there is some evidence that acetazolamide may help prevent HM episodes [10]. The exact mechanism of action is unknown, but it is believed that acetazolamide may alter the local pH around the P/Q calcium channel by inhibiting carbonic anhydrase and improving ion channel performance. Usually, the medication is taken twice a day at a dose of approximately 250 mg [7,11]. The use of vasoconstrictive drugs in HM is debated due to the increasing concern for aggravation of aura and exacerbation of HM attacks; therefore, triptans are historically contraindicated. Few anti-epileptic medicines, such as topiramate, have also been effective in some patients for long-lasting aura symptoms; however, more evidence is required to establish their effectiveness [3].

The mainstay treatment of HM is done with abortive and preventive therapy; however, severe attacks may require prompt hospitalization with additional precautions such as fluid balance and food intake [2,3]. If the patient develops fever and convulsions, it should be managed symptomatically. Administering 20 mg of furosemide intravenously to patients experiencing recurrent migrainous aura resulted in the disappearance of their aura symptoms, as observed in a study by Rozen [12]. Suggesting the potential of furosemide in averting chronic migrainous auras triggered by cortical spreading depression. In another study, Rozen identified prochlorperazine and magnesium sulfate as potential therapies for managing chronic migrainous aura [13]. Furthermore, Centonze et al. reported in their study that intravenous administration of 0.4 mg of naloxone improved two patients with migraines, effectively halting the neurological effects of the migraines without relieving pain [14].

The above treatment modality has also been found effective for HM, and non-selective Ca2+ blockers prophylaxis can be effective; however, severe attacks cannot be prevented [8].

Clinical recommendations and long-term follow-up

Proper case history can lead to an accurate diagnosis. History should include the correct order of symptoms and type of onset, duration of symptoms, family history, and proper clinical examination. Diagnosis should made according to ICHD-3 criteria [2,6]. Neuroimaging may or may not be typical since the appearance of radiological findings depends on the imaging time, and signs may disappear after the resolution of the neurological deficit [3]. Patients diagnosed with SHM showed evolution in their families, and SHM changed into FHM, suggesting SHM can precede FHM in a few patients' families [15]. Management of FMH requires interval treatment to reduce the frequency of episodes and progression into life-threatening neurological symptoms during episodes. Repeat clinical examination, MRI, EEG, neuropsychological testing, and assessment should be done periodically [8,15]. Treatment with non-selective CCB is effective; however, severe attacks cannot be prevented entirely [8].

## Conclusions

This report underscores the intricate process of diagnosing and managing HM, accentuating the pivotal role of thorough clinical assessment and meticulous history-taking. By delving deeply into the patient's medical background and symptomatology, healthcare providers can swiftly recognize the distinctive features of HM, paving the way for tailored treatment approaches. This report also emphasizes the long-term follow-up of patients diagnosed with FHM and their first-degree relatives. Prophylactic therapy in FHM cases can decrease the frequency of attacks in such patients. However, early identification facilitated by history-taking enables prompt initiation of targeted interventions in new cases, thus optimizing patient outcomes and mitigating the burdensome effects of this neurological condition. Moreover, the comprehensive evaluation enabled by history-taking empowers healthcare providers to discern potential warning signs, such as recent head trauma or focal neurological deficits, prompting timely investigation and intervention when necessary. By prioritizing early recognition and individualized care plans, healthcare teams can effectively alleviate symptoms and improve the overall quality of life for individuals grappling with HM.
